# Human Glial-Restricted Progenitor Transplantation into Cervical Spinal Cord of the SOD1^G93A^ Mouse Model of ALS

**DOI:** 10.1371/journal.pone.0025968

**Published:** 2011-10-05

**Authors:** Angelo C. Lepore, John O'Donnell, Andrew S. Kim, Timothy Williams, Alicia Tuteja, Mahendra S. Rao, Linda L. Kelley, James T. Campanelli, Nicholas J. Maragakis

**Affiliations:** 1 Department of Neuroscience, Thomas Jefferson University Medical College, Philadelphia, Pennsylvania, United States of America; 2 Department of Neurology, Johns Hopkins University School of Medicine, Baltimore, Maryland, United States of America; 3 Life Technologies, Carlsbad, California, United States of America; 4 Department of Internal Medicine, University of Utah Health Sciences Center, Salt Lake City, Utah, United States of America; 5 Q Therapeutics, Salt Lake City, Utah, United States of America; 6 Department of Neurology, University of Utah, Salt Lake City, Utah, United States of America; Seattle Children's Research Institute, United States of America

## Abstract

Cellular abnormalities are not limited to motor neurons in amyotrophic lateral sclerosis (ALS). There are numerous observations of astrocyte dysfunction in both humans with ALS and in SOD1^G93A^ rodents, a widely studied ALS model. The present study therapeutically targeted astrocyte replacement in this model via transplantation of human Glial-Restricted Progenitors (hGRPs), lineage-restricted progenitors derived from human fetal neural tissue. Our previous findings demonstrated that transplantation of rodent-derived GRPs into cervical spinal cord ventral gray matter (in order to target therapy to diaphragmatic function) resulted in therapeutic efficacy in the SOD1^G93A^ rat. Those findings demonstrated the feasibility and efficacy of transplantation-based astrocyte replacement for ALS, and also show that targeted multi-segmental cell delivery to cervical spinal cord is a promising therapeutic strategy, particularly because of its relevance to addressing respiratory compromise associated with ALS. The present study investigated the safety and *in vivo* survival, distribution, differentiation, and potential efficacy of hGRPs in the SOD1^G93A^ mouse. hGRP transplants robustly survived and migrated in both gray and white matter and differentiated into astrocytes in SOD1^G93A^ mice spinal cord, despite ongoing disease progression. However, cervical spinal cord transplants did not result in motor neuron protection or any therapeutic benefits on functional outcome measures. This study provides an *in vivo* characterization of this glial progenitor cell and provides a foundation for understanding their capacity for survival, integration within host tissues, differentiation into glial subtypes, migration, and lack of toxicity or tumor formation.

## Introduction

ALS is a motor neuron disorder characterized by relatively rapid degeneration of upper and lower motor neurons, with death from respiratory failure normally occurring 2–5 years following diagnosis [1]. Approximately 90–95% of ALS cases are sporadic in nature, with 20% of the remaining familial cases linked to various point mutations in the Cu/Zn superoxide dismutase 1 (SOD1) gene [2]. Transgenic mice [3], [4], [5] and rats [6], [7], [8] carrying ALS-associated mutant human SOD1 genes recapitulate many features of the human disease.

Despite the relative selectivity of motor neuron cell death, animal and tissue culture models of familial ALS suggest that non-neuronal cells significantly contribute to neuronal dysfunction and death [9]. CNS astrocytes outnumber their neuronal counterparts approximately ten-fold and play key roles in adult CNS homeostasis, including the vast majority of synaptic glutamate uptake, maintenance of extracellular potassium and nutrient support of neurons [10]. Multiple properties of spinal cord and brain astrocytes are compromised in ALS, and these changes often precede clinical disease onset [11]. Initial evidence for an astrocyte contribution to ALS came from studies of humans [12], [13] and rodent ALS models [6] indicating dysfunction and large decreases in levels of the primary astrocyte glutamate transporter, GLT1 (EAAT2 in human), in areas of motor neuron loss. Confirmation of a role for non-neuronal cells in modulating mutant SOD1-induced pathological changes in neighboring motor neurons came from studies of chimeras of mutant SOD1-expressing cells [14] and from mice in which mutant SOD1 expression was selectively reduced in astrocytes [15]. These studies highlight the important role of astrocyte-motor neuron interactions in the etiology of ALS.

Regardless of whether astrocyte dysfunction is a cause of disease or a consequence of neuronal death, altered astrocyte physiology results in further susceptibility to motor neuron loss and contributes to disease progression. We previously reported that targeted enrichment of normal astrocytes in SOD1^G93A^ rat cervical spinal cord via intraspinal transplantation of rodent-derived Glial-Restricted Progenitors (GRPs) promoted focal motor neuron protection, delayed decline in respiratory function and extension in disease progression [16]. Efficacy was partly mediated by transplant-based replacement of astrocyte GLT1, presumably via restoration of extracellular glutamate homeostasis by preventing ALS-associated loss of GLT1. GRPs were specifically transplanted around cervical spinal cord respiratory motor neuron pools (phrenic motor neurons), the principal cells innervating the diaphragm whose dysfunction precipitates death in ALS patients [17], [18] and animals expressing mutant SOD1 [19], [20]. Collectively, these findings indicated the feasibility and efficacy of transplantation-based astrocyte replacement and showed that targeted multi-segmental cell delivery to cervical spinal cord is a promising therapeutic strategy for slowing focal motor neuron loss, particularly because of its relevance to addressing respiratory compromise associated with ALS.

The overall goal of the present study was to extend this work to an analogous class of human GRPs [21], [22]. We sought to demonstrate safety and therapeutic efficacy of transplantation of hGRPs into the cervical spinal cord ventral horn of the SOD1^G93A^ mouse model of ALS, as well as to extensively characterize the long-term *in vivo* cell fate of hGRPs in the ALS spinal cord.

## Materials and Methods

### Ethics Statement

Brain tissue from fetal cadavers of gestational age from 17 to 24 weeks is used as the starting material for isolation and purification procedures of hGRPs. Tissue is procured by Procurement Specialists employed by Advanced Bioscience Resources (ABR; Alameda CA; FEIN 3005208435) following Donor ID and Informed Consent SOPs and Donor Medical Record Review procedures. ABR has contractual agreements with physicians and medical facilities or clinics in the United States wherein pregnancy terminations take place, which allow ABR personnel to be present in the medical facility at the time of the surgical procedures. When a patient has decided to proceed with a pregnancy termination, she signs a clinic-generated informed consent to that effect. After she has made that decision and signed the surgical consent, the patient is presented with information regarding the opportunity to donate fetal tissue and to allow a peripheral blood sample to be taken from her, for infectious disease testing or to identify specific markers in the blood. If she desires to participate in donation, she signs an additional ABR-generated consent for that purpose, which is presented and witnessed by clinic staff. Clinic staff inform on-site ABR personnel regarding the patients' wishes for participation or declination. If the patient agrees to participate, ABR personnel wait for the surgical termination to be completed, and then proceed with tissue identification and procurement. If suitable fetal or other POC tissue is identified for distribution, ABR's certified phlebotomists may then acquire a peripheral blood sample from the participating patient by performing venipuncture, or by utilizing the clinic-inserted IV. The historical average is four tissues per month (over the past two years).

The Johns Hopkins Institutional Review Board has concluded that studies with surgical materials are exempted from Human Subjects Approval pursuant to Federal Register 46.101 Exemption Number 4. Research described will involve the preparation of surgical samples in which “information is recorded by the investigator in such a manner that subjects cannot be identified directly or through identifiers linked to the subjects”. The application (#NA_00021112) was reviewed by JHM IRB. This obtaining of cells was funded through the Maryland Stem Cell Research Act of 2006. The IRB determined on August 7, 2008 that the project qualified for an exemption determination under federal regulations at 45 CFR 46.101(a).

The Johns Hopkins Animal Care and Use Committee (ACUC) approved the research protocol #MO10M449 for the transplantation studies of these cells on Dec 9, 2010 (expires Dec 9, 2011).

### Human Glial-Restricted Progenitors (hGRPs)

#### Derivation and Selection of Human Glial-Restricted Progenitors (hGRPs)

hGRPs (also referred to as Q-Cells^®^) were derived as previously described [21], [22]. Briefly, fetal forebrain (17–24 weeks gestational age) was mechanically and enzymatically dissociated, followed by positive selection via magnetic-activated cell sorting with the glial progenitor-specific cell-surface antigen, A_2_B_5_.

#### Culturing of hGRPs

Following selection, hGRPs were cultured *in vitro* on polyornithine-coated flasks [DMEM-F12 with L-glutamine and 15.0 mM 4-(2-hydroxyethyl)-1-piperazineethanesulfonic acid (Life Technologies; Carlsbad, CA), 1X N1 (Sigma-Aldrich; St. Louis, MO), 0.01% human serum albumin (Baxter; Deerfield, IL), 20.0 ng/mL bFGF (Peprotech; Rocky Hill, NJ), 10.0 ng/mL PDGF-AA (Peprotech)] for 20 days and subsequently frozen [0.5X medium, 1X ProFreeze non-animal origin freezing medium (Lonza BioWhittaker; Basel, Switzerland), 7.5% DMSO (Sigma-Aldrich)]. Frozen aliquots were thawed and immediately prepared for injections as described below [16].

### Cell Transplantation

#### Experimental Design

Three transplantation experiments were conducted. In **Cohort #1**, 50–60 day old SOD1^G93A^ mice were transplanted with “low dose” of hGRPs (Q-Cells; n = 16 SOD1^G93A^ mice) or human fibroblasts (hFs; ScienCell, Human Dermal Fibroblasts - fetal; n = 22), and were immunesuppressed via daily Sub-Q injections of CSA. In **Cohort #2**, 50–60 day old SOD1^G93A^ mice again received “low dose” of hGRPs (n = 20) or hFs (n = 20), but were immunosuppressed via daily I.P. injections of FK-506 (Tacrolimus)/Rapamycin (Sirolimus). In **Cohort #3**, 50–60 day old SOD1^G93A^ mice received “high dose” of hGRPs (n = 11) or hFs (n = 12), and were again immunosuppressed via daily I.P. injections of FK-506/Rapamycin. Refer to Table 1 for summary of experimental design.

**Table 1:** Summary of Experimental Findings: Human Glial-Restricted Progenitor Transplantation into Cervical Spinal Cord of the SOD1^G93A^ Mouse Model of ALS.

	Cohort #1		Cohort #2		Cohort #3	
**Immune Suppression**	Cyclosporin A (Sub-Q)10 mg/kg daily		FK-506/Rapamycin (I.P.)1 mg/kg each daily		FK-506/Rapamycin (I.P.)1 mg/kg each daily	
**Cell Dose**	“Low Dose” Transplantation50,000 cells/site4 sites/spinal cord		“Low Dose” Transplantation50,000 cells/site4 sites/spinal cord		“High Dose” Transplantation150,000 cells/site4 sites/spinal cord	
**Age at Txn (Days)**	50–60	50–60	50–60	50–60	50–60	50〉60
**Cell Type**	Human Fibroblasts	Q Cells	Human Fibroblasts	Q Cells	Human Fibroblasts	Q Cells
**Survival (Days)**	119.0+/− 1.9	121.0+/− 2.2	125.4+/− 2.3	127.9+/− 1.9	119.0+/− 1.9	122.5+/14.1
**Hindlimb Onset (Days)**	94.5+/− 2.3	94.4+/− 3.6	106.8+/− 1.7	108.6+/− 2.1	99.4+/− 14.4	101.2+/− 13.3
**Forelimb Onset (Days)**	111.7+/− 2.4	112.3+/− 2.8	117.5+/− 0.1	117.4+/− 1.7	112.9+/− 12.9	106.6+/− 15.7
**Duration (Days)**	24.5+/− 2.5	26.3+/− 3.3	18.1+/− 2.7	18.8+/− 1.7	15.3+/− 3.2	15.0+/− 2.8
**CMAPs (mV)**	ND	ND	4.3+/− 0.7	4.5+/− 0.6	2.7+/− 0.4	3.3+/− 0.7
**Cervical Motor Neurons/Section**	ND	ND	5.6+/− 0.1	6.1+/− 0.4	2.98+/− 0.1	2.95+/− 0.1
**n**	22	16	20	20	12	11
	p>0.05 for all analyses		p>0.05 for all analyses		p>0.05 for all analyses	

#### Preparation of hGRPs for Transplantation

hGRPs and hFs were suspended (in basal medium) at a concentration of either 2.5×10^4^ cells/µL (Experiments 1 and 2) or 7.5×10^4^ cells/uL (Experiment 3). After the completion of the transplantation session, cell viability was assessed using the trypan blue assay and was always found to be greater than 75%.

#### Human GRP Transplantation

Immune suppressed animals received transplants at 50–60 days of age. Each mouse received 4 grafts (bilaterally at C4 and C5) of 5.0×10^4^ cells/site (Experiments 1 and 2) or 1.5×10^5^ cells/site (Experiment 3) (in 2 µL basal media) into ventral horn. Briefly, cells were delivered using a 10 µL Hamilton Gastight syringe with an attached 30-gauge 45° beveled needle (Hamilton; Reno, NV). The injection pipette was secured to a manual micromanipulator (World Precision Instruments; Sarasota, FL) attached to an 80° tilting base [16]. The tip was lowered to a depth of 0.75 mm below the surface of the cord and was held in place for 2 minutes before and after cell injection. Cells were delivered under the control of a microsyringe pump controller (World Precision Instruments) at a rate of 0.5 µL/minute.

#### Immune suppression

Animals were immune suppressed by subcutaneous administration of cyclosporine A (10 mg/kg; Sandoz Pharmaceuticals, East Hanover, NJ) or by intraperitoneal administration of FK-506/Rapamycin (1 mg.kg/each; LC Laboratories; Woburn, MA) daily beginning three days before grafting and continuously until sacrifice.

#### SOD1^G93A^ mice

Transgenic mice carrying the human SOD1 gene with the G93A mutation were used (B6SJL-Tg(SOD1*G93A)1Gur/J: Stock # 002726) [23], [24]. Male and female mice were obtained from The Jackson Laboratory (Bar Harbor, ME), and maintained as an in-house colony. On average, untreated SOD1^G93A^ mutants developed hindlimb disease onset on average at 95–105 days of age, subsequently developed forelimb onset on average at 110–120 days of age, and reached disease endstage at approximately 120–125 days of age. For all studies, equal numbers of males and females were included in all groups, and animals from the same litter were distributed amongst groups.

#### Care and Treatment of Animals

All procedures was conducted in strict accordance with the guidelines set by the European Communities Counsel Directive (November 24th, 1986), the NIH Guide for the Care and Use of Laboratory Animals, the Guidelines for the Use of Animals in Neuroscience Research and the Johns Hopkins University IACUC, and measures were taken to minimize any potential pain or animal discomfort. Mice were housed at standard temperature (21°C) and in a light controlled environment with ad libitum access to the food and water, and were maintained in racks of ventilated cages located in the same room. In order to avoid dehydration, Aqua-Jel packs were provided when animals started to show disease symptoms.

### Behavioral and Electrophysiological Analyses

#### Hindlimb and Forelimb Grip Strength

Animal weighing and all behavioral data collection began 1 week prior to transplantation, and was conducted twice weekly until end-stage. Hind- and forelimb muscle grip strengths were separately determined using a “Grip Strength Meter” (DFIS-2 Series Digital Force Gauge; Columbus Instruments, OH) [16]. Grip strength testing was performed by allowing the animals to grasp a thin bar attached to the force gauge. This was followed by pulling the animal away from the gauge until the hind- or forelimbs released the bar. This provides a value for the force of maximal grip strength. The force measurements were recorded in three separate trials, and the averages were used in analyses.

#### Disease Onset

Hind- and forelimb disease onsets were assessed individually for each mouse by a 20.0% loss in hind- or forelimb grip strength relative to each animal's own baseline grip strength level [16].

#### Survival/Endstage Analysis

To determine disease endstage in a reliable and ethical fashion, endstage was defined by the inability of mice to right themselves within 30 seconds when placed on their sides.

#### Disease Duration

Overall onset of disease was determined by hindlimb grip strength onset because hindlimb deficits were the first clinical symptoms observed in the vast majority of mice. Disease duration was measured as time between hindlimb disease onset and disease endstage [16]. All SOD1^G93A^ animals were included in overall survival, disease onset and grip strength analyses; however, mice that displayed forelimb onset prior to hindlimb onset (approximately 10% of SOD1^G93A^ mice) were excluded from disease duration analysis, as well as from analysis of the delay of forelimb onset following hindlimb onset.

#### Compound Muscle Action Potential (CMAP) Recordings

Under anesthesia, phrenic nerve conduction studies were performed with stimulation (0.5 ms single stimulus; 1 Hz supramaximal pulses) at the neck via near nerve needle electrodes placed 0.5 cm apart along the phrenic nerve [19], [20]. Recording was obtained via a surface strip along the costal margin, and CMAP amplitude was measured baseline to peak. Recordings across the nerve segment were made using an ADI Powerlab 8SP stimulator and BioAMP amplifier (AD Instruments: Colorado Springs, CO), followed by computer assisted data analysis (Scope 3.5.6; ADI). Distal motor latency of evoked potentials includes duration of nerve conduction between stimulating and recording electrodes plus time of synaptic transmission. CMAPs were collected at only a single time point (120 days of age).

### Histological and Biochemical Analyses

#### Tissue Processing

Animals were sacrificed at 2 and 4 weeks post-transplantation, at 120 days of age, or at disease endstage by transcardial perfusion with 0.3% saline, followed by ice-cold 4% paraformaldehyde (Fisher Scientific; Pittsburgh, PA). Spinal cords were removed from the animal, followed by cryoprotection in 30% sucrose (Fisher)/.1 M phosphate buffer at 4°C for 3 days. The tissue was embedded in OCT (Fisher), fast frozen with dry ice, and stored at −80°C until processed. Spinal cord tissue blocks were cut in the sagittal or transverse planes at 30 µm thicknesses. Sections were collected on glass slides and stored at −20°C until analyzed. Subsets of spinal cord slices were collected in PBS for free-floating histochemistry.

#### Quantification of Transplant Survival and Migration

HuNA (human nuclear antigen; Millipore; Temecula, CA; monoclonal; 1∶400) was used to selectively identify transplant-derived human cells (both hGRPs and hFs). Total numbers of HuNA^+^/DAPI^+^ hGRPs (or hFs) were quantified in every 6^th^ section throughout the extent of transplanted spinal cord at disease endstage [16]. Cell numbers from all sections were summed and multiplied by 6 to obtain the total number of surviving cells. Values were expressed as a percentage of the total number of cells transplanted. hFs were derived from fetal human dermis. Survival of hFs following transplantation was also assessed with HuNA.

#### Differentiation and Proliferation Markers

Nestin (1∶1000; monoclonal; Pharmingen; San Diego, CA) was used to identify undifferentiated NPCs and GRPs [25]. GRPs were identified using A_2_B_5_ (1∶500; Millipore) [26]. Neurons were identified using βIIITubulin (1∶100; polyoclonal; Covance; Princeton, NJ) [16]. Motor neurons were identified with ChAT (1∶500; monoclonal; Millipore). Astrocytes were identified using pan-GFAP (1∶400; polyclonal; Dako; Glostrup, Denmark) or human-specific GFAP (1∶5,000; monoclonal; Dako) [23]. Cells of the oligodendrocyte lineage were identified using Olig2 (1∶200; polyclonal; Millipore) [27]. Proliferating cells were identified with Ki67 (1∶400; monoclonal; Lab Vision Corp; Fremont, CA) [25]. Pre-synaptic terminals were identified with synapsin (1∶100; polyclonal; BD Biosciences; Sparks, MD). Microglial cells were detected via Iba1 (1∶200; polyclonal; Wako; Richmond, VA) [16]. GLT1 expression by hGRPs was assessed via immunohistochemistry with GLT1 antibody (carboxyl terminus-directed antibody; 1∶500; polyclonal) (**Table 2**) [6]. Samples were incubated for 2 hours at room temperature with goat anti-mouse and goat anti-rabbit secondary antibodies (1∶200; Jackson ImmunoResearch; West Grove, PA) conjugated to rhodamine or FITC. Samples were counterstained with DAPI (1∶1000; Sigma-Aldrich) to identify nuclei, and cover-slipped with anti-fade mounting media (Fluorosave, CN Biosciences; La Jolla, CA). Slides were subsequently stored at 4°C. Images were acquired on either a Zeiss fluorescence microscope using a Photometric Sensys KAF-1400 CCD camera (Roper Scientific; Trenton, NJ) or on a Zeiss laser confocal microscope. Images were analyzed using either Metamorph or Zeiss confocal software. Adobe Photoshop 7.0 (Adobe; San Jose, CA) was used to prepare figures.

**Table 2:** Antibody Details.

Antibody	Target of Recognition	Manufacturer	Species	Dilution
Nestin	Undifferentiated Neural Precursor	Pharmingen	Mouse Monoclonal	1∶1,000
A_2_B_5_	Glial-Restricted Precursors	Millipore	Mouse Monoclonal	1∶500
βIIITubulin	Neurons	Covance	Rabbit Polyoclonal	1∶100
ChAT	Motor Neurons	Millipore	Mouse Monoclonal	1∶500
GFAP	Astrocytes	Dako	Rabbit Polyoclonal	1∶400
Olig2	Oligodendrocyte Lineage	Millipore	Rabbit Polyoclonal	1∶200
Ki67	Proliferating Cells	Lab Vision	Rabbit Polyoclonal	1∶400
Synapsin	Pre-synaptic Terminals	BD Bio	Rabbit Polyoclonal	1∶100
Iba1	Microglia	Wako	Rabbit Polyoclonal	1∶200
GLT1	Astrocyte Glutamate Transporter	Rothstein Lab	Rabbit Polyoclonal	1∶500

#### Quantification of Transplant Differentiation and Proliferation

The proportions of transplant-derived neurons (NeuN), astrocytes (pan-GFAP), cells of the oligodendrocyte lineage (Olig2), microglia (Iba1) and actively proliferating cells (Ki67) were expressed as a percentage of the total number of transplanted cells counted at specific locations along the cervical spinal cord relative to sites of injection [16]. In order to quantify double-labeling of HuNA with lineage-specific (pan-GFAP, Olig2, NeuN, Iba1) and proliferation (Ki67) markers, double-labeled sagittal sections containing the entire cervical enlargement, as well as additional rostral and caudal spinal cord tissue, were imaged at 20× magnification using Zeiss AxioVision software. Images were analyzed in AxioVision at specific distances from the injection sites. For each animal (n = 6 mice), 6 different 20× images were analyzed at each specific distance (3 in gray matter, 3 in white matter. In each image, every HuNA^+^ cell with a matching DAPI^+^ nucleus was assessed for co-expression of the lineage-specific marker, and these data were pooled from the 3 images to determine the value at each distance for each animal.

#### Motor Neuron Survival

The cervical (C4–C6) spinal cord was serially sectioned transversely (30 µm) and stained with cresyl violet (Sigma-Aldrich) to quantify motor neuron numbers. Motor neurons were counted in every 7th section at 20× magnification in order to avoid repetitive counting. Only motor neurons with a clearly identifiable nucleus and nucleolus, a cell soma over 100 µm^2^ and located within the ventral horn were counted at a 200-fold magnification, and the final counts were multiplied by seven to give an estimate of total cell numbers [16].

#### Western Analysis

Aliquots of homogenized cord samples from C5 were separately subjected to SDS-polyacrylamide gel electrophoresis. Blots were probed with antibody specific for GLT-1 (1∶5,000) [16]. Immunoreactivity was visualized by enhanced chemiluminescence, quantified densitometrically using Quantity One v4.0 software (BioRad; Hercules, CA), and normalized to actin.

### Statistical Analyses

Kaplan-Meier analysis of the SOD1^G93A^ mice was conducted using the statistical software Sigmastat (SAS Software) to analyze survival, disease onset and duration data. Weight and grip strength results were analyzed via ANOVA. In some cases, Student *t-test* was performed to compare data between groups of animals. All data are presented as mean ± S.E.M., and significance level was set at p≤0.05.

## Results

Human cells were transplanted into the cervical spinal cord of SOD1^G93A^ mice. Three separate transplantation experiments were conducted. In Cohort #1, 50–60 day old SOD1^G93A^ mice were transplanted with “low dose” of hGRPs (5.0×10^4^ cells/site; 4 sites) (n = 16) or unmodified human fibroblasts (hFs; n = 22), and were immune suppressed via daily subcutaneous injections of CSA. In Cohort #2, 50–60 day old SOD1^G93A^ mice again received “low dose” of hGRPs (5.0×10^4^ cells/site; 4 sites) (n = 20) or hFs (n = 20 mice), but were immune suppressed via daily I.P. injections of FK-506 (Tacrolimus)/Rapamycin. In Cohort #3, 50–60 day old SOD1^G93A^ mice received “high dose” of hGRPs (1.5×10^5^ cells/site; 4 sites) (n = 11) or hFs (n = 12), and were again immune suppressed via daily I.P. injections of FK-506/Rapamycin. Refer to Table 1 for summary of experimental design.

### Cohort #1: Human GRPs showed poor survival with cyclosporine immunosuppression

**Figure 1:**
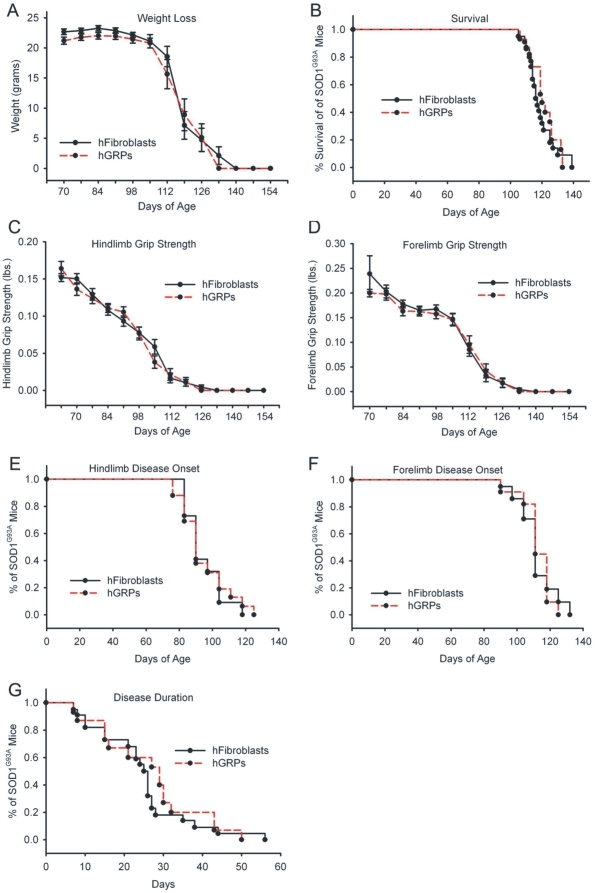
Cohort #1: “low dose” hGRP transplantation in combination with CSA did not promote functional efficacy. Compared to control transplantation of hFs, “low dose” hGRP transplantation in combination with CSA-based immune suppression neither accelerated nor slowed progression of disease in SOD1^G93A^ mice. Unchanged outcome measures included weight loss (**A**), overall survival (**B**), declines in hindlimb (**C**) and forelimb (**D**) grip strength, hindlimb (**E**) and forelimb (**F**) disease onsets, and disease duration (**G**).

Compared to control transplantation of hFs, “low dose” hGRP transplantation in combination with CSA-based immune suppression neither accelerated nor slowed progression of disease in SOD1^G93A^ mice (see **Table 1** and **Figure 1** for summary of findings). Unchanged outcome measures included weight loss (**Fig. 1A**), overall survival (**Fig. 1B**), declines in hindlimb (**Fig. 1C**) and forelimb (**Fig. 1D**) grip strength, hindlimb (**Fig. 1E**) and forelimb (**Fig. 1F**) disease onsets, and disease duration (**Fig. 1G**). This study demonstrates the feasibility and safety of a cervical cellular transplantation approach in this mouse model of ALS.

Human GRP transplants did not survive at disease endstage in SOD1^G93A^ mice with CSA-based immune suppression at 10 mg/kg administered I.P. (data not shown). Because of the effectiveness of an alternative immune suppression regimen, the combination of FK-506 and Rapamycin, in supporting hGRP transplant survival in SOD1^G93A^ mouse spinal cord (pilot study data not shown), FK-506/Rapamycin was used in subsequent studies to examine therapeutic efficacy and *in vivo* transplant fate of hGRPs.

### Cohort #2: Human GRP characterization with FK-506 and Rapamycin immunosuppression

#### Cell Survival and Migration

**Figure 2:**
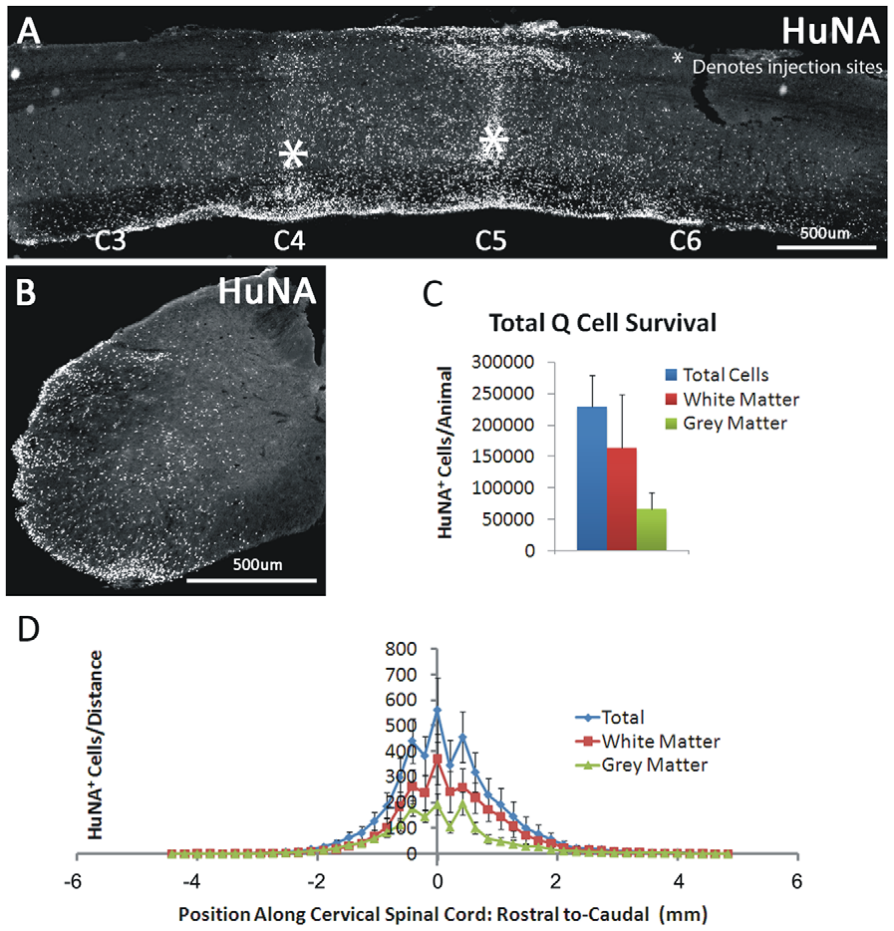
Cohort #2: hGRPs in combination with FK-506/Rapamycin survived and migrated in SOD1^G93A^ cervical spinal cord. Unlike the lack of hGRP survival observed in SOD1^G93A^ mice with CSA-based immune suppression, HuNA^+^ hGRP transplant-derived cells survived until disease endstage (up to 3 months post-transplantation) with FK-506/Rapamycin (**A**–**D**). Cells survived in both white and gray matter regions; however, a larger number of hGRP-derived cells were located in the white matter (**C**–**D**). On average, greater than 200,000 total cells survived in each spinal cord at endstage (**C**). HuNA^+^ cells also migrated both rostrally and caudally throughout the gray and white matter of the cervical spinal cord, up to distances of 0.6 cm from the injection sites; however, the vast majority of cells were located within 2 mm rostral and caudal of the injection sites (**D**). Scale bars: 500 µm.

**Figure 3:**
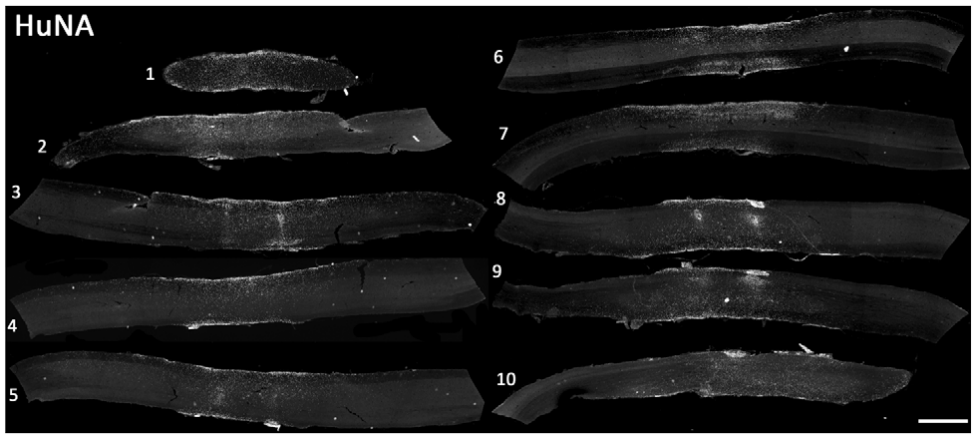
Cohort #2: hGRPs with FK-506/Rapamycin occupied a significant portion of the cervical spinal cord in SOD1^G93A^ mice. Human GRP transplant-derived cells (from 4 injection sites: bilaterally at C4 and C5) dispersed to occupy a significant portion of the cervical spinal cord, as demonstrated by the sagittal series of HuNA-stained sections from a single animal (slices are approximately 200 µm apart). Scale bar: 500 µm.

Unlike the lack of hGRP survival observed in SOD1^G93A^ mice with CSA-based immune suppression, HuNA^+^ hGRP transplant-derived cells survived until disease endstage (up to 3 months post-transplantation) with FK-506/Rapamycin (**Fig. 2A**–**D**). Cells survived in both white and gray matter regions; however, a larger number of hGRP-derived cells were located in the white matter (**Fig. 2C**–**D**). On average, greater than 200,000 total cells survived in each spinal cord at endstage, slightly more than the total number of cells injected at all four sites combined (**Fig. 2C**). HuNA^+^ cells also migrated both rostrally and caudally throughout the gray and white matter of the cervical spinal cord, up to distances of 6.0 mm from the injection sites; however, the vast majority of cells were located within 2 mm rostral and caudal of the injection sites (**Fig. 2D**). Human GRP transplant-derived cells (from 4 injection sites: bilaterally at C4 and C5) dispersed to occupy a significant portion of the cervical spinal cord, as demonstrated by the sagittal series of HuNA-stained sections from a single animal shown in **Figure 3** (slices are approximately 200 µm apart). Unlike hGRPs, very low survival of hFs was observed at disease endstage (data not shown), which may be due to the ectopic nature of a human dermal fibroblast transplant into host rodent neural tissue.

#### Proliferation

**Figure 4:**
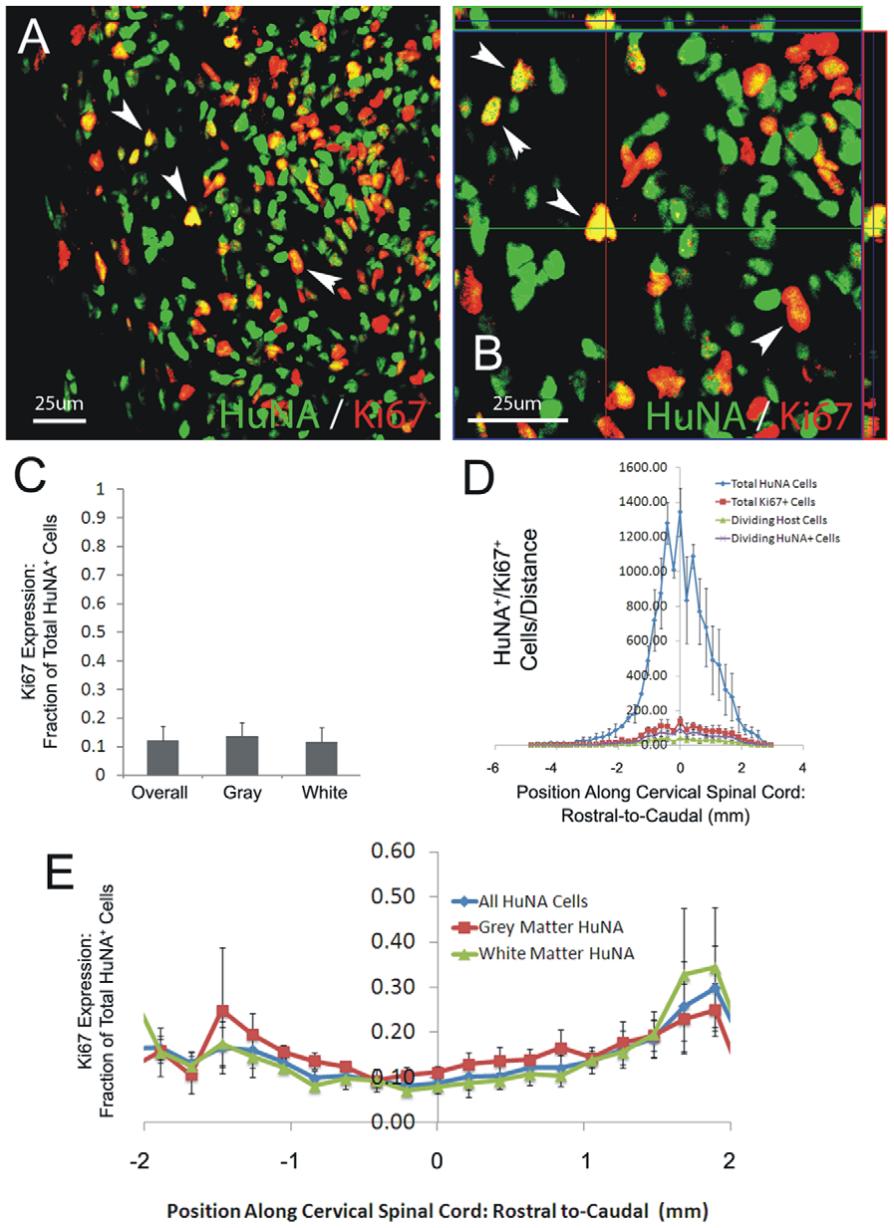
Cohort #2: hGRPs with FK-506/Rapamycin continued to proliferate in SOD1^G93A^ cervical spinal cord. Approximately 10% of transplant-derived HuNA^+^ cells continued to divide at disease endstage, as determined by co-expression of the proliferation marker, Ki67 (**A**–**B**). No differences were noted between gray and white matter (**C**–**D**), and the percentage of proliferating cells slightly increased with distance from injection sites (**E**). Scale bars: 25 µm.

Approximately 10% of transplant-derived HuNA^+^ cells continued to divide at disease endstage, as determined by co-expression of the proliferation marker, Ki67 (**Fig. 4A–B**). No differences were noted between gray and white matter (**Fig. 4C–D**), and the percentage of proliferating cells slightly increased with distance from injection sites (**Fig. 4E**), suggesting that a larger proportion of migrating cells remained in an undifferentiated state compared to cells closer to the injection sites. While the proportion of cells that continued to divide remained small, these data suggest that a small amount of continued proliferation over several months likely contributed to the impressive degree of cell survival observed (i.e. numbers of cells equal to or greater than originally transplanted).

#### Neuronal Differentiation

**Figure 5:**
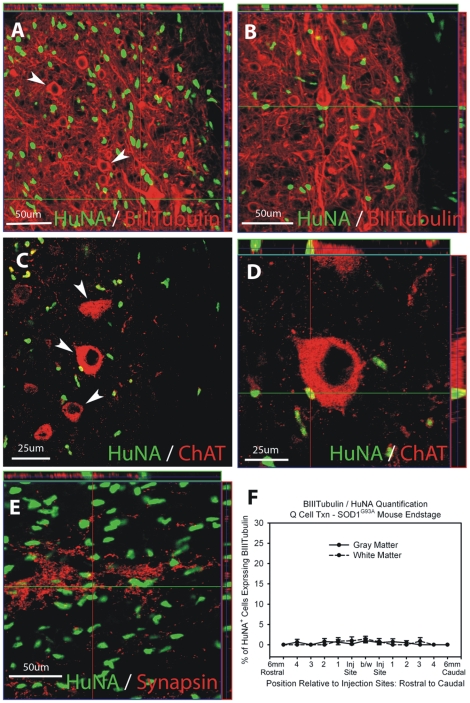
Cohort #2: hGRPs with FK-506/Rapamycin did not differentiate into neurons in SOD1^G93A^ cervical spinal cord. HuNA^+^ hGRP transplant-derived cells did not differentiate into βIII-Tubulin^+^ neurons (**A**–**B**). At disease endstage, less than 1% of all HuNA^+^ cells co-expressed βIII-Tubulin (**F**). HuNA^+^ cells spatially interacted with ChAT^+^ motor neurons (**C**–**D**) and synapsin^+^ pre-synaptic sites (**E**) within the spinal cord of SOD1^G93A^ mice. Scale bars: A–B, E 50 µm; C-D 25 µm.

HuNA^+^ hGRP transplant-derived cells did not differentiate into βIII-Tubulin^+^ neurons (**Fig. 5A**–**B**). At disease endstage, less than 1% of all HuNA^+^ cells co-expressed βIII-Tubulin (**Fig. 5F**). HuNA^+^ cells spatially interacted with ChAT^+^ motor neurons (**Fig. 5C**–**D**) and synapsin^+^ pre-synaptic sites (**Fig. 5E**) within the spinal cord of SOD1^G93A^ mice.

#### Astrocyte Differentiation

**Figure 6:**
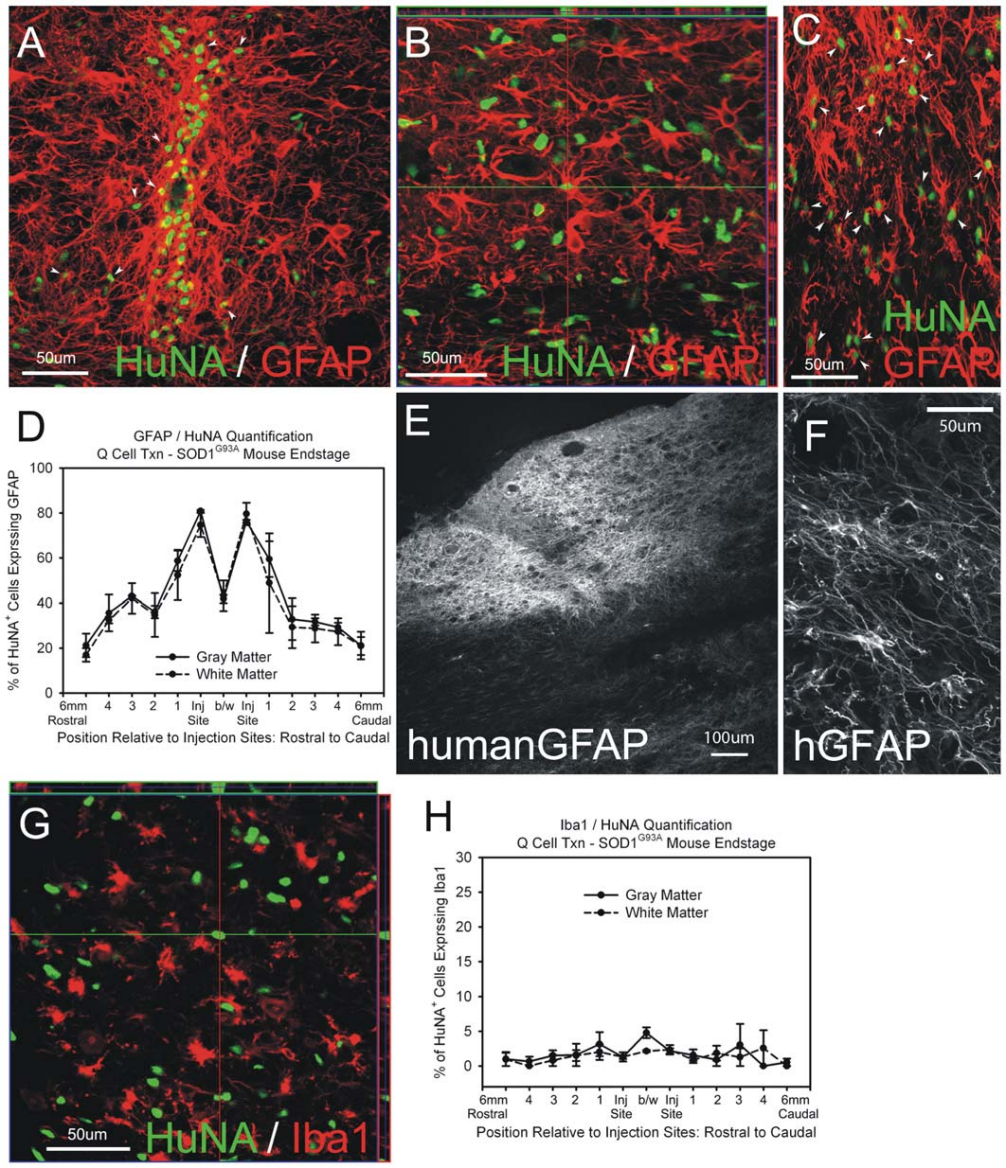
Cohort #2: hGRPs with FK-506/Rapamycin differentiated into astrocytes in SOD1^G93A^ cervical spinal cord. Transplanted HuNA^+^ hGRPs differentiated into GFAP^+^ astrocytes by disease endstage in gray matter both near the injection site (**A**) and at distances up to 6 mm (**B**: image represents region with migrating cells approximately 3 mm caudal to the injection site), as well as in white matter (**C**). At disease endstage, approximately 50–80% of all HuNA^+^ cells co-expressed the astrocyte marker GFAP at sites within 1 mm rostral or caudal of the injection sites, regardless of whether cells were found in gray (**A**) or white (**C**) matter (**D**). This percentage decreased at greater distances from the injection sites (**B**, **D**). In addition to the pan-GFAP marker, transplanted-derived astrocytes were labeled with the human-specific GFAP marker (**E**–**F**). Only a minor fraction (approximately 1%) of HuNA^+^ cells co-expressed the microglial marker, Iba1 (**G**–**H**). Arrowheads denote HuNA/GFAP double-labeled cells in panels **A** and **C**. Scale bars: 50 µm.

Transplanted HuNA^+^ hGRPs differentiated into GFAP^+^ astrocytes by disease endstage in gray matter both near the injection site (**Fig. 6A**) and at distances up to 6 mm (**Fig. 6B**: image represents region with migrating cells approximately 3 mm caudal to the injection site), as well as in white matter (**Fig. 6C**). At disease endstage, approximately 50–80% of all HuNA^+^ cells co-expressed the astrocyte marker GFAP at sites within 1 mm rostral or caudal of the injection sites, regardless of whether cells were found in gray or white matter (**Fig. 6D**). This percentage decreased at greater distances from the injection sites. Across the entire spinal cord, 57.1 +/− 0.02% of all HuNA^+^ cells differentiated into GFAP astrocytes, with little difference between gray and white matter regions (gray matter: 58.8 +/− 0.02%; white matter: 56.1 +/− 0.10%). Only 25–40% of HuNA^+^ hGRP transplant-derived cells differentiated into GFAP^+^ astrocytes when analyzed at 3 weeks post-transplantation (not shown). In addition to the pan-GFAP marker, transplanted-derived astrocytes were labeled with the human-specific GFAP marker (**Fig. 6E**–**F**). Only a minor fraction (approximately 1%) of HuNA^+^ cells co-expressed the microglial marker, Iba1 (**Fig. 6G**–**H**).

**Figure 7:**
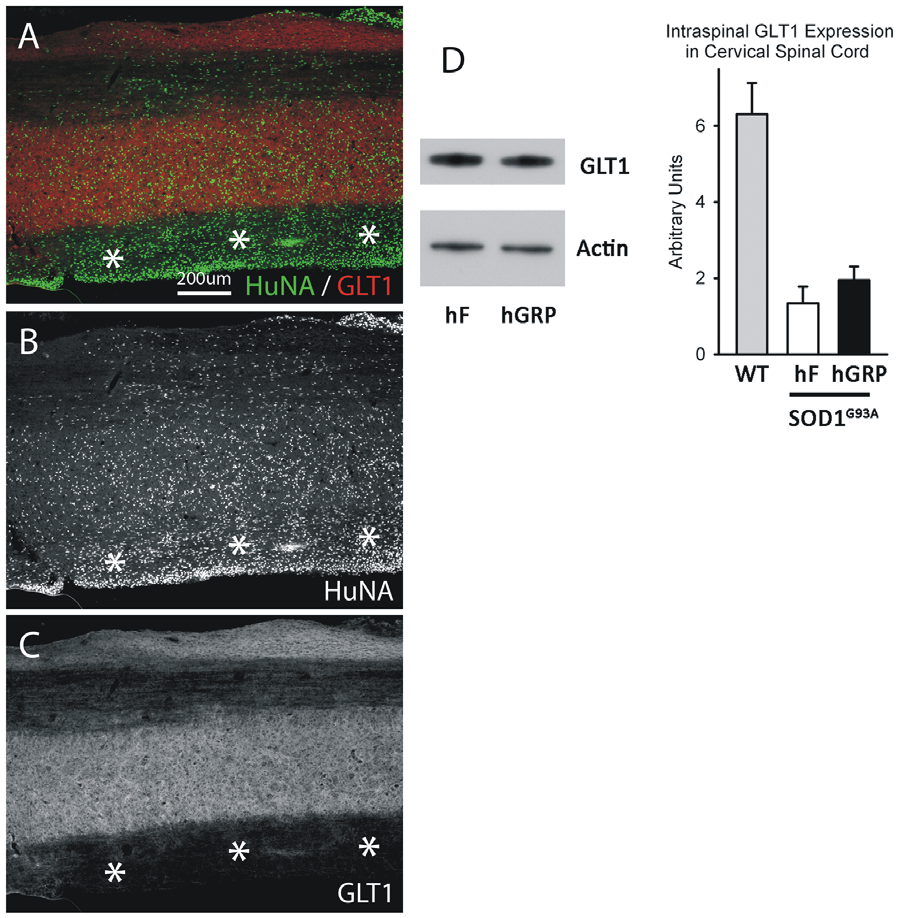
Cohort #2: hGRPs did not express the astrocyte glutamate transporter GLT1 in SOD1^G93A^ cervical spinal cord. Immunohistochemical analysis human GRP transplant-derived cells does not reveal expression of the astrocyte glutamate transporter, GLT1, in the SOD1^G93A^ spinal cord at disease endstage (**A**–**C**). Intraspinal levels of GLT1 were measured using Western blotting of whole cervical spinal cord segments at C5. hGRPs did not slow the loss of intraspinal GLT1 protein levels compared to hF controls (**D**). Asterisks denote regions with large numbers of transplant-derived cells, but no GLT1 expression. Scale bars: 200 µm.

Human GRP transplant-derived cells did not appear to express the major astrocyte glutamate transporter, GLT1, in the SOD1^G93A^ spinal cord at disease endstage (**Fig. 7A**–**C**). Intraspinal levels of GLT1 were measured using Western blotting of whole cervical spinal cord segments at C5. Human GRPs did not slow the loss of intraspinal GLT1 protein levels compared to hF controls (**Fig. 7D**).

#### Oligodendrocyte Lineage Differentiation

**Figure 8:**
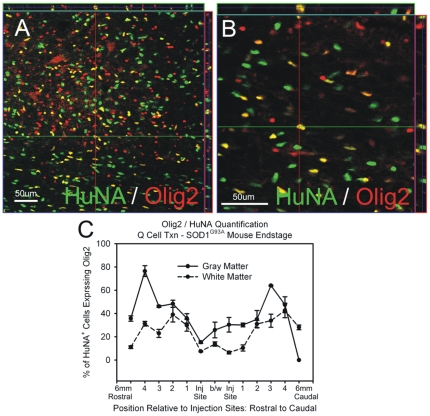
Cohort #2: hGRPs with FK-506/Rapamycin differentiated towards the oligodendrocyte lineage in SOD1^G93A^ cervical spinal cord. At disease endstage, HuNA^+^ cells co-labeled with the oligodendrocyte lineage marker, Olig2 (**A**–**B**). Close to the injection sites, approximately 10–30% of the HuNA^+^ cells expressed Olig2, in both gray and white matter regions (**C**). This percentage was higher at greater distances from the injection sites. Scale bars: 50 µm.

At disease endstage, HuNA^+^ cells co-labeled with the oligodendrocyte lineage marker, Olig2 (**Fig. 8A**–**B**). However, it is possible that Olig2 also marks glial precursor cells destined to differentiate into astrocytes [27]. Close to the injection sites, approximately 10–30% of the HuNA^+^ cells expressed Olig2, in both gray and white matter regions (**Fig. 8C**). This percentage was higher at greater distances from the injection sites, which taken together with the inverse correlation of GFAP expression percentage with distance (Fig. 6B), may suggest that a GRP progeny biased to become an oligodendrocyte (OPC) has greater migratory ability than a GRP progeny biased to become an astrocyte (APC).

#### Lack of Phenotypic Efficacy

**Figure 9:**
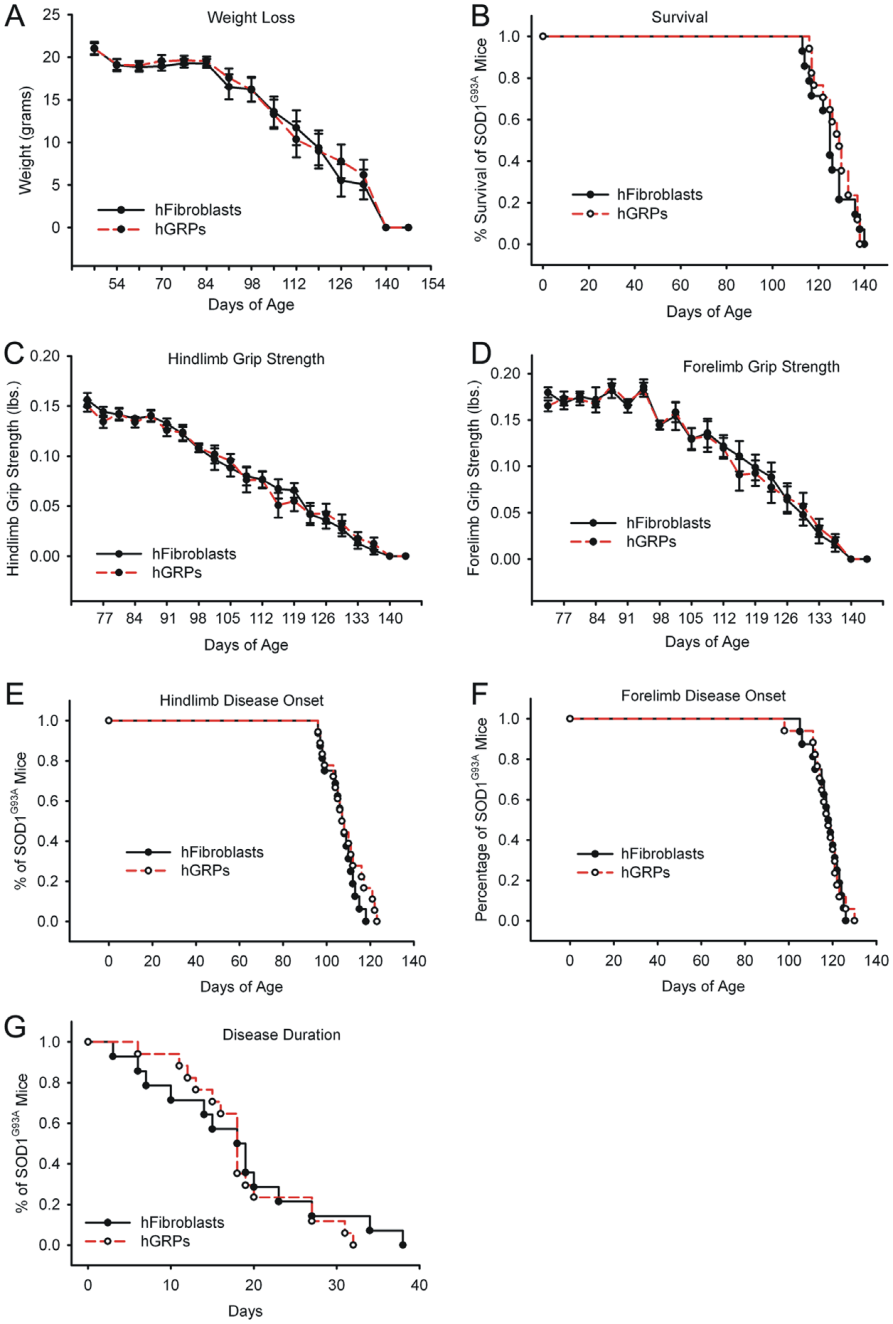
Cohort #2: “low dose” hGRP transplantation with FK-506/Rapamycin did not promote functional efficacy. Compared to control transplantation of hFs, “low dose” hGRP transplantation in combination with FK-5-6/Rapamycin-based immune suppression neither accelerated nor slowed progression of disease in SOD1^G93A^ mice. Unchanged outcome measures included weight loss (**A**), overall survival (**B**), declines in hindlimb (**C**) and forelimb (**D**) grip strength, hindlimb (**E**) and forelimb (**F**) disease onsets, and disease duration (**G**).

Despite robust survival and the significant differentiation of hGRPs into astrocytes throughout the cervical spinal cord of SOD1^G93A^ mice, “low dose” hGRP transplantation in combination with FK-506 and Rapamycin-based immune suppression neither accelerated nor slowed progression of disease in SOD1^G93A^ mice (see **Table 1** and **Figure 9** for summary of findings) when compared to control transplantation of hFs. Unchanged outcome measures included weight loss (**Fig. 9A**), overall survival (**Fig. 9B**), declines in hindlimb (**Fig. 9C**) and forelimb (**Fig. 9D**) grip strength, hindlimb (**Fig. 9E**) and forelimb (**Fig. 9F**) disease onsets, and disease duration (**Fig. 9G**). Taken together with the robust hGRP survival and distribution documented above, these findings further document the safety of the surgical transplantation approach, as well as the safety of persistent hGRP grafts *in vivo*.

#### Lack of Neuroprotection

**Figure 10:**
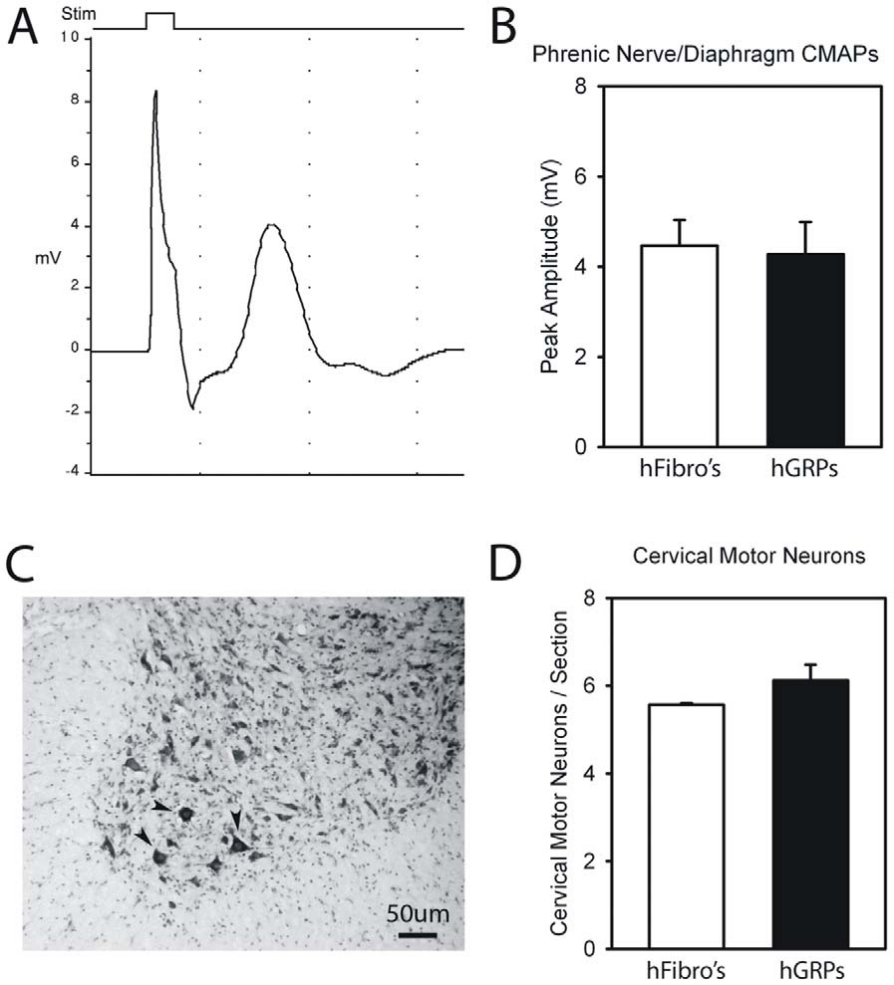
Cohort #2: “low dose” hGRP transplantation with FK-506/Rapamycin did not protect respiratory function or cervical motor neurons. All SOD1^G93A^ animals had significantly reduced phrenic CMAP amplitudes at 120 days of age (**A**), demonstrating that SOD1^G93A^-mediated disease results in compromised respiratory function; however, there was no significant difference between hGRP and hF groups (**B**). Cervical motor neurons (denoted by arrowheads) were counted throughout the cervical enlargement in cresyl violet stained sections (**C**). There was no difference in cervical motor neuron survival between mice receiving hGRP and hF transplants at disease endstage (**D**). Scale bars: 50 µm.

To evaluate the ability of hGRPs to preserve respiratory function in SOD1^G93A^ animals, mice receiving hGRP (n = 5) or hF (n = 6) transplants were tested at 120 days of age (+/− 3 days) for peak compound muscle action potentials (CMAP) amplitudes (**Fig. 10A**) in one hemi-diaphragm following ipsilateral phrenic nerve stimulation, an electrophysiological assay of respiratory function [19], [20]. Pre-symptomatic SOD1^G93A^ mice had peak CMAP amplitudes of 7.0–8.0mV (not shown). All SOD1^G93A^ animals had significantly reduced CMAP amplitudes at 120 days of age, demonstrating that SOD1^G93A^-mediated disease results in compromised respiratory function; however, there was no significant difference between experimental groups (**Fig. 10B**).

This lack of efficacy as assessed by behavioral and respiratory functional outcome measures was paralleled by a lack of neuroprotection of cervical spinal cord motor neurons. Cervical motor neurons were counted throughout the cervical enlargement in cresyl violet stained sections (**Fig. 10C**). There was no difference in cervical motor neuron survival between mice receiving hGRP and hF transplants at disease endstage (**Fig. 10D**; n = 4/group).

### Cohort #3: High Dose Human GRPs Transplantation with FK-506 and Rapamycin Immunosuppression

#### Cell Survival and Migration

**Figure 11:**
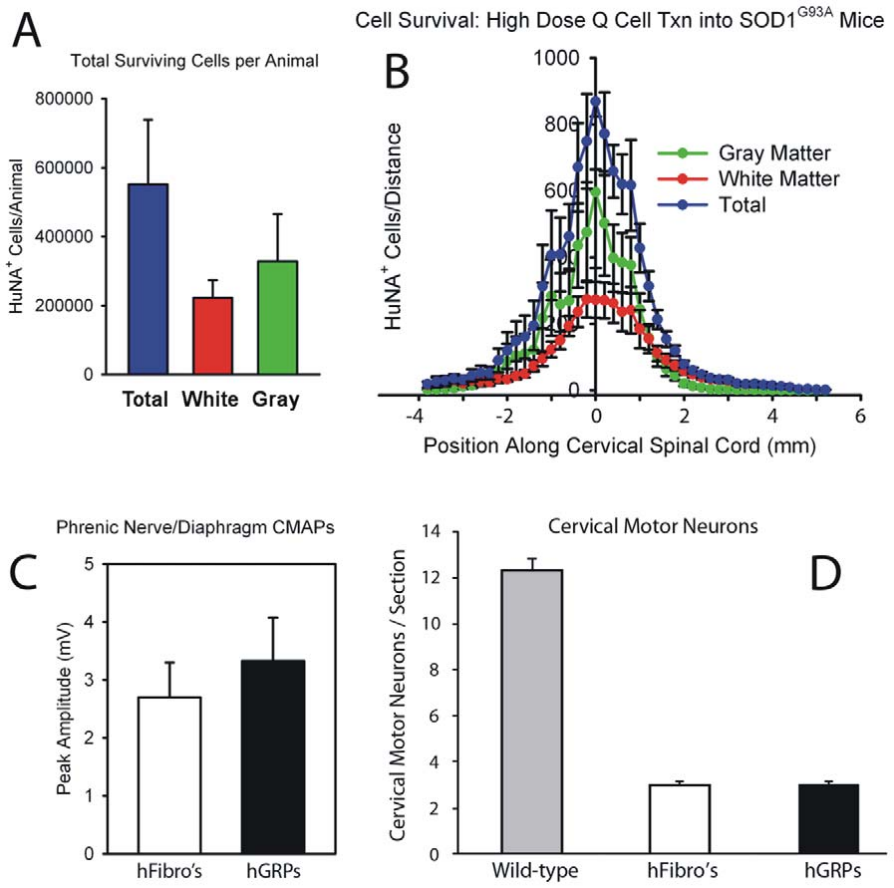
Cohort #3: “high dose” hGRP transplantation with FK-506/Rapamycin did not protect respiratory function or cervical motor neurons. Greater cell survival was found with the “high dose” transplants compared to “low dose” transplants, with a greater proportion of cells localizing to gray matter than white matter (**A**). Transplant-derived cells migrated from injection sites up to distances of 6 mm (**B**). All SOD1^G93A^ animals had significantly reduced phrenic CMAP amplitudes at 130 days of age, however, there was no significant difference between hGRP and hF groups (**C**). There was no difference in cervical motor neuron survival between mice receiving hGRP and hF transplants at disease endstage (**D**).

Similar to “low dose” transplantation with FK-506/Rapamycin, hGRP transplants robustly survived until disease endstage. The patterns of survival, localization and migration (**Fig. 11B**), as well as differentiation (data not shown), were similar to those documented for “low dose” transplant fate. As expected, greater cell numbers were found at endstage with the “high dose” transplants (**Fig. 11A**).

Interestingly, the bias seen in Cohort 2 (low dose) toward white matter residence of graft progeny (**Fig. 2C**–**D**) was reversed in the high dose cohort (**Fig. 11A**–**B**). It is worth noting that the absolute number of hGRP progeny present in white matter at both doses was comparable, possibly suggesting a saturation point for exogenously grafted cells in the white matter microenvironment.

#### Lack of Phenotypic Efficacy

Given the lack of efficacy observed with hGRP cell transplantation despite survival and astrocyte differentiation, we evaluated the therapeutic efficacy of an increased “dose” of transplanted hGRPs, three times the cell number used in Cohort 1 and 2. Cells were delivered using the exact same protocol as described above.

**Figure 12:**
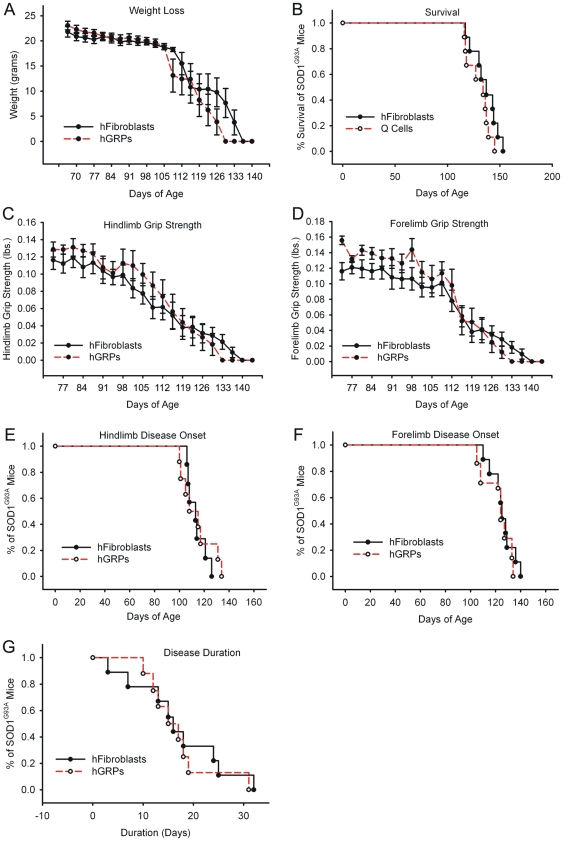
Cohort #3: “high dose” hGRP transplantation with FK-506/Rapamycin did not promote functional efficacy. Compared to control “high dose” transplantation of hFs, “high dose” hGRP transplants in combination with FK-506 and Rapamycin-based immune suppression neither accelerated nor slowed progression of disease in SOD1^G93A^ mice. Unchanged outcome measures included weight loss (**A**), overall survival (**B**), declines in hindlimb (**C**) and forelimb (**D**) grip strength, hindlimb (**E**) and forelimb (**F**) disease onsets, and disease duration (**G**).

Compared to control “high dose” transplantation of hFs, “high dose” hGRP transplants in combination with FK-506 and Rapamycin-based immune suppression neither accelerated nor slowed progression of disease in SOD1^G93A^ mice (see **Table 1** and **Figure 12** for summary of findings). Unchanged outcome measures included weight loss (**Fig. 12A**), overall survival (**Fig. 12B**), declines in hindlimb (**Fig. 12C**) and forelimb (**Fig. 12D**) grip strength, hindlimb (**Fig. 12E**) and forelimb (**Fig. 12F**) disease onsets, and disease duration (**Fig. 12G**).

#### Lack of Neuroprotection

All SOD1^G93A^ animals had significantly reduced CMAP amplitudes at 130 days of age; however, there was no significant difference between experimental groups (**Fig. 11C**; n = 5/group). There was also no difference in cervical motor neuron survival between mice receiving hGRP and hF transplants at disease endstage (**Fig. 11D**; n = 5/group).

## Discussion

One of the greatest challenges in potentially using cell replacement therapy for ALS is the progressive nature of the disease. This progression has made the concept of using motor neuron progenitors for reinervation and regeneration a limiting factor. Current technologies do not currently exist to magnify axon outgrowth to outpace a neurodegenerative disease where survival is 2–5 years following a diagnosis [28]. With this in mind, cell therapeutic strategies have turned to non-neuronal cells (including astrocytes, but also myocytes, oligodendrocytes, microglia, and mesenchymal stem cells, amongst others) for their potential in providing motor neuron protection and slowing disease progression.

We have previously reported that wild-type rodent-derived GRPs (rGRPs) transplanted into the cervical spinal cords of SOD1^G93A^ rats differentiated into astrocytes and provided neuroprotection resulting in phenotypic improvement in these ALS animals [16]. The present study investigated the safety and *in vivo* survival, distribution, differentiation, and potential efficacy of clinically relevant analogous human Glial-Restricted Progenitors (hGRPs) [21], [22]. We report that hGRP transplants survived and distributed throughout much of the cervical spinal cord of SOD1^G9A3^ mice and differentiated into astrocytes, but did not provide a phenotypic sparing of function.

At disease endstage, approximately 50–80% of hGRPs differentiated into astrocytes close to the injection sites, while this percentage sharply dropped with increasing distances. We observed differentiation of hGRPs into cells of the oligodendrocyte lineage as well. These data suggest that while the majority of hGRPs differentiate into astrocytes in this *in vivo* paradigm, these cells continue to maintain their capacity for differentiation into other glial phenotypes, as has been described previously [29]. Interestingly, Walczak and colleagues showed that hGRP transplantation into an inflammatory demyelinated adult rodent spinal cord also showed significant differentiation of hGRPs into GFAP^+^ astrocytes, with only minimal oligodendrocyte differentiation [30]. Whether the propensity of hGRPs to differentiate primarily into astrocytes reflects the immunosuppression regimen chosen (both the current study and Walczak et al. used FK-506 and Rapamycin), the nature of the lesion, the proximity of the cells to the transplant site, or the natural propensity of these cells to differentiate into GFAP^+^ astrocytes requires further investigation.

Human GRP-derived cells also continued to proliferate when assessed at endstage. While 10% of hGRP-derived cells continued to proliferate, instances of tumor formation were never found at endstage (up to 3 months post-transplantation). Gross pathological examination of other organs outside the CNS did not demonstrate heterotopic engraftment. The absence of either tumor formation within the CNS or heterotopic engraftment in tissues outside the CNS is important in establishing the safety of such cells with regard to their translational capacity for ALS treatment.

Our data suggest that survival of transplanted cells critically depends on the successful implementation of the appropriate immune suppression regimen. hGRPs showed poor long-term survival using the calcineurin inhibitor, cyclosporine A. This necessitated the combination of FK-506 (tacrolimus) and Rapamycin (sirolimus), a regimen that targets calcineurin- or mTOR-dependent inhibition of T cell activation, respectively [31] (these non-specific immune suppression compounds likely exert effects on other pathways as well). Autologous derivation of cells for transplantation via patient-specific technologies such as induced Pluripotent Stem (iPS) cells [32] may eventually obviate the need for chronic immune suppression of transplant recipients in a clinical setting, but emerging data also suggest that iPS cells may also have immunogenic potential [33]. Therefore, the study of cell-based therapy for transplantation may require parallel analyses of varying immunosuppression regimens in order to determine the most efficacious options for cell survival.

A number of differences in the experimental paradigms between the present work and our previous study utilizing rGRPs transplanted into SOD1 animal models has made direct comparisons with the potential utility of hGRPs in managing ALS limited. These include, first and foremost, the use of a shorter-lived SOD1^G93A^ mouse in the current study, as compared with the longer-living SOD1^G93A^ rat model that we previously used. Other differences include the immunosuppression regimen, number of injection sites in the spinal cord (4 in the SOD1^G93A^ mouse and 6 in the previous SOD1^G93A^ rat study: due to significant species spinal cord size differences), and fewer numbers of transplanted cells used in the current study (6×10^5^ total cells in current study's “high-dose” group compared to 9×10^5^ in the previous SOD1^G93A^ rat study). A number of studies have shown that analogous rodent and human stem/progenitor cells differ in a variety of properties, including culturing conditions, gene and protein expression, proliferation rate and propensity to differentiate into various mature lineages [34], [35]. These differences may account for the lack of efficacy reported in this study, arising for example from functional differences in the way that xenografted cells alter host physiology relative to allografts or closely related (rat/mouse) grafts due to inter-species incompatibility.

Previous studies have transplanted human neural stem cells, some genetically modified to release GDNF, into the spinal cords of SOD1^G93A^ rats. Following transplantation, those neural stem cells releasing GDNF were able to protect motor neurons in regions where transplanted cells integrated near host motor neurons. Similar to our current observations, those neural stem cells also retained immature cellular markers at sites distant from transplantation and did not have any therapeutic benefit - presumably because of continued distal denervation at the neuromuscular junction [36]. Other studies using human neural progenitor cells expressing GDNF or IGF-1 in the mouse SOD1 model also showed neuroprotection, but no effect on survival [37].

The present study was conducted to extensively characterize the *in vivo* fate of human GRPs derived from the human fetal CNS and to evaluate the therapeutic efficacy of a clinically-relevant neural precursor cell type in an animal model of ALS. This approach targets therapy to diaphragmatic function, and is therefore an important strategy given the central role played by respiratory dysfunction in the ultimate death of patients [17], [18]. While there are numerous classes of undifferentiated neural precursors [38], as well as growing interest in clinically-relevant technologies such as iPS cells [32], we have shown that transplantation of lineage-restricted progenitors provides a valuable tool for achieving robust survival and integration, as well as targeted and efficient differentiation into specific glial phenotypes *in vivo*.

Future studies with an eye towards clinical translation of this approach may include an increase in the number of cells transplanted in order to calculate both a “dose” of cells for efficacy and also to establish a maximum tolerated “dose” of cells before toxicity becomes a limiting factor. Increasing the number of transplant sites for achieving delivery of cells to additional regions of the spinal cord may also result in improved efficacy [39]. *In vitro* manipulations prior to transplantation to increase the yield of hGRP-derived astrocytes may result in improved host motor neuron survival. Because this approach is aimed at neuroprotection rather than neuronal replacement, reconstitution of spinal cord astrocytes, once optimized, may be a valuable approach to cellular-based therapeutics.

## References

[1] Bruijn LI, Miller TM, Cleveland DW (2004). Unraveling the mechanisms involved in motor neuron degeneration in ALS.. Annu Rev Neurosci.

[2] Rosen DR, Siddique T, Patterson D, Figlewicz DA, Sapp P (1993). Mutations in Cu/Zn superoxide dismutase gene are associated with familial amyotrophic lateral sclerosis.. Nature.

[3] Bruijn LI, Becher MW, Lee MK, Anderson KL, Jenkins NA (1997). ALS-linked SOD1 mutant G85R mediates damage to astrocytes and promotes rapidly progressive disease with SOD1-containing inclusions.. Neuron.

[4] Gurney ME, Pu H, Chiu AY, Dal Canto MC, Polchow CY (1994). Motor neuron degeneration in mice that express a human Cu,Zn superoxide dismutase mutation.. Science.

[5] Wong PC, Pardo CA, Borchelt DR, Lee MK, Copeland NG (1995). An adverse property of a familial ALS-linked SOD1 mutation causes motor neuron disease characterized by vacuolar degeneration of mitochondria.. Neuron.

[6] Howland DS, Liu J, She Y, Goad B, Maragakis NJ (2002). Focal loss of the glutamate transporter EAAT2 in a transgenic rat model of SOD1 mutant-mediated amyotrophic lateral sclerosis (ALS).. Proc Natl Acad Sci U S A.

[7] Matsumoto A, Okada Y, Nakamichi M, Nakamura M, Toyama Y (2006). Disease progression of human SOD1 (G93A) transgenic ALS model rats.. J Neurosci Res.

[8] Nagai M, Aoki M, Miyoshi I, Kato M, Pasinelli P (2001). Rats expressing human cytosolic copper-zinc superoxide dismutase transgenes with amyotrophic lateral sclerosis: associated mutations develop motor neuron disease.. J Neurosci.

[9] Ilieva H, Polymenidou M, Cleveland DW (2009). Non-cell autonomous toxicity in neurodegenerative disorders: ALS and beyond.. J Cell Biol.

[10] Pekny M, Nilsson M (2005). Astrocyte activation and reactive gliosis.. Glia.

[11] Maragakis NJ, Rothstein JD (2006). Mechanisms of Disease: astrocytes in neurodegenerative disease.. Nat Clin Pract Neurol.

[12] Rothstein JD, Martin LJ, Kuncl RW (1992). Decreased glutamate transport by the brain and spinal cord in amyotrophic lateral sclerosis.. N Engl J Med.

[13] Rothstein JD, Van Kammen M, Levey AI, Martin LJ, Kuncl RW (1995). Selective loss of glial glutamate transporter GLT-1 in amyotrophic lateral sclerosis.. Ann Neurol.

[14] Clement AM, Nguyen MD, Roberts EA, Garcia ML, Boillee S (2003). Wild-type nonneuronal cells extend survival of SOD1 mutant motor neurons in ALS mice.. Science.

[15] Yamanaka K, Chun SJ, Boillee S, Fujimori-Tonou N, Yamashita H (2008). Astrocytes as determinants of disease progression in inherited amyotrophic lateral sclerosis.. Nat Neurosci.

[16] Lepore AC, Rauck B, Dejea C, Pardo AC, Rao MS (2008). Focal transplantation-based astrocyte replacement is neuroprotective in a model of motor neuron disease.. Nat Neurosci.

[17] Haverkamp LJ, Appel V, Appel SH (1995). Natural history of amyotrophic lateral sclerosis in a database population. Validation of a scoring system and a model for survival prediction.. Brain.

[18] Mitsumoto H, Chad DA, Pioro EP (1998). Amyotrophic lateral sclerosis..

[19] Lepore AC, Tolmie C, O'Donnell J, Wright MC, Dejea C (2010). Peripheral hyperstimulation alters site of disease onset and course in SOD1 rats.. Neurobiol Dis.

[20] Llado J, Haenggeli C, Pardo A, Wong V, Benson L (2006). Degeneration of respiratory motor neurons in the SOD1 G93A transgenic rat model of ALS.. Neurobiol Dis.

[21] Campanelli JT, Sandrock RW, Wheatley W, Xue H, Zheng J (2008). Expression profiling of human glial precursors.. BMC Dev Biol.

[22] Sandrock RW, Wheatley W, Levinthal C, Lawson J, Hashimoto B (2010). Isolation, characterization and preclinical development of human glial-restricted progenitor cells for treatment of neurological disorders.. Regen Med.

[23] Lepore AC, Dejea C, Carmen J, Rauck B, Kerr DA (2008). Selective ablation of proliferating astrocytes does not affect disease outcome in either acute or chronic models of motor neuron degeneration.. Exp Neurol.

[24] Lepore AC, Haenggeli C, Gasmi M, Bishop KM, Bartus RT (2007). Intraparenchymal spinal cord delivery of adeno-associated virus IGF-1 is protective in the SOD1G93A model of ALS.. Brain Res.

[25] Lepore AC, Fischer I (2005). Lineage-restricted neural precursors survive, migrate, and differentiate following transplantation into the injured adult spinal cord.. Exp Neurol.

[26] Lepore AC, Neuhuber B, Connors TM, Han SS, Liu Y (2006). Long-term fate of neural precursor cells following transplantation into developing and adult CNS.. Neuroscience.

[27] Han SS, Kang DY, Mujtaba T, Rao MS, Fischer I (2002). Grafted lineage-restricted precursors differentiate exclusively into neurons in the adult spinal cord.. Exp Neurol.

[28] Papadeas ST, Maragakis NJ (2009). Advances in stem cell research for Amyotrophic Lateral Sclerosis.. Curr Opin Biotechnol.

[29] Maragakis NJ, Dietrich J, Wong V, Xue H, Mayer-Proschel M (2004). Glutamate transporter expression and function in human glial progenitors.. Glia.

[30] Walczak P, All AH, Rumpal N, Gorelik M, Kim H (2011). Human glial-restricted progenitors survive, proliferate, and preserve electrophysiological function in rats with focal inflammatory spinal cord demyelination.. Glia.

[31] Yan J, Xu L, Welsh AM, Chen D, Hazel T (2006). Combined immunosuppressive agents or CD4 antibodies prolong survival of human neural stem cell grafts and improve disease outcomes in amyotrophic lateral sclerosis transgenic mice.. Stem Cells.

[32] Takahashi K, Tanabe K, Ohnuki M, Narita M, Ichisaka T (2007). Induction of pluripotent stem cells from adult human fibroblasts by defined factors.. Cell.

[33] Zhao T, Zhang ZN, Rong Z, Xu Y (2011). Immunogenicity of induced pluripotent stem cells.. Nature.

[34] Chandran S, Compston A, Jauniaux E, Gilson J, Blakemore W (2004). Differential generation of oligodendrocytes from human and rodent embryonic spinal cord neural precursors.. Glia.

[35] Ray J, Gage FH (2006). Differential properties of adult rat and mouse brain-derived neural stem/progenitor cells.. Mol Cell Neurosci.

[36] Suzuki M, McHugh J, Tork C, Shelley B, Klein SM (2007). GDNF secreting human neural progenitor cells protect dying motor neurons, but not their projection to muscle, in a rat model of familial ALS.. PLoS One.

[37] Park S, Kim HT, Yun S, Kim IS, Lee J (2009). Growth factor-expressing human neural progenitor cell grafts protect motor neurons but do not ameliorate motor performance and survival in ALS mice.. Exp Mol Med.

[38] Lepore AC, Maragakis NJ (2007). Targeted stem cell transplantation strategies in ALS.. Neurochem Int.

[39] Xu L, Shen P, Hazel T, Johe K, Koliatsos VE (2011). Dual transplantation of human neural stem cells into cervical and lumbar cord ameliorates motor neuron disease in SOD1 transgenic rats.. Neurosci Lett.

